# Long‐term prognosis of enteral feeding and parenteral nutrition in a population aged 75 years and older: a population‐based cohort study

**DOI:** 10.1186/s12877-020-02003-x

**Published:** 2021-01-28

**Authors:** Yukio Tsugihashi, Manabu Akahane, Yasuhiro Nakanishi, Tomoya Myojin, Shinichiro Kubo, Yuichi Nishioka, Tatsuya Noda, Shuichiro Hayashi, Shiori Furihata, Tsuneyuki Higashino, Tomoaki Imamura

**Affiliations:** 1 Department of Public Health, Health Management and Policy, Nara Medical University, 840 Shijo-Cho, 634-8521 Kashihara, Nara Japan; 2 Department of Health and Welfare Services, National Institute of Public Health, 2-3-6 Minami, 351-0197 Wako-shi, Saitama, Japan; 3 Healthcare and Wellness Division, Mitsubishi Research Institute, Inc, 10-3, Nagatacho 2-Chome, Chiyoda-Ku, 100-8141 Tokyo, Japan

**Keywords:** Enteral feeding, Parenteral nutrition, Gastrostomy, Nasogastric tube feeding, Intravascular hyperalimentation, Mortality, Health insurance claims, Japan

## Abstract

**Background:**

Enteral feeding and parenteral nutrition (PN) using gastrostomy (GS) and a nasogastric tube feeding (NGT) and PN should be initiated for older patients based on their prognoses. This study aimed to investigate the long-term prognosis of patients aged ≥75 years who underwent enteral feeding via GS and NGT as well as PN.

**Methods:**

A population-based cohort study was conducted using Japan’s universal health insurance claims in the Nara Prefecture. This study enrolled 3,548 patients aged ≥75 years who received GS (*N*=770), NGT (*N*=2,370), and PN (*N*=408) during hospital admissions between April 2014 and March 2016. The GS group was further categorized into secondary GS (*N*=400) with preceding NGT or PN within 365 days and primary GS (*N*=370) without preceding NGT or PN groups. In the secondary GS group, 356 (96%) patients received NGT (versus PN). The outcome was mortality within 730 days after receiving GS, NGT, and PN. Cox regression analyses in cases with or without malignant diseases, adjusted for sex, age, comorbidity, and hospital type, were performed to compare mortality in the groups.

**Results:**

Of the 3,548 participants, 2,384 (67%) died within 730 days after the initiation of GS and NGT and PN. The 2-year mortality rates in the secondary GS, primary GS, NGT, and PN groups were 58%, 66%, 68%, and 83% in patients without malignancies and 67%, 71%, 74%, and 87% in those with malignancies, respectively. In the non-malignant group, Cox regression analysis revealed that secondary GS (hazard ratio (HR) = 0.43, 95% CI: 0.34–0.54), primary GS (HR = 0.51, 95% CI: 0.40–0.64), and NGT (HR = 0.71, 95% CI: 0.58-0.87) were statistically significantly associated with lower mortality compared with PN.

**Conclusions:**

Approximately 58% to 87% patients aged ≥75 years died within 730 days after initiation of nutrition through GS, NGT, or PN. Patients with non-malignant diseases who received secondary GS exhibited better 2-year prognosis than those who received NGT or PN. Healthcare professionals should be aware of the effectiveness and limitations of enteral feeding and PN when considering their initiation.

## Background

Enteral feeding and parenteral nutrition are usually administered enterally with gastric tubes and parenterally with peripheral or central venous access [1]. Enteral feeding can improve the nutritional status and prognoses of patients with dysphagia due to cerebral stroke [2, 3], head injury [4], and neuromuscular diseases [5, 6]. Since the development of percutaneous endoscopic gastrostomy (PEG) in 1980 [7], gastrostomy (GS) has been widely used as a common procedure for long-term enteral nutrition in older patients [8, 9]. Although nasogastric tube feeding (NGT) is a time-proven technique for enteral nutrition, it should not be used for more than 4–6 weeks because of complications or poor adherence to treatment [10, 11]. In addition, parenteral nutrition (PN) can be recommended when patients have an intestinal failure or malignant bowel obstruction [12, 13]. Previous studies reported that in-hospital mortalities associated with enteral feeding were lower than those associated with PN among non-cancer patients and patients with eating disorders [14, 15]. A study on the prognoses after PEG in older patients in Japan indicated that the 30-day mortality was 10–12%, the 1-year mortality was 30–33%, and more than 50% of the patients survived for over 2 years [16–18]. However, to date, year-long mortalities after initiating nutrition through GS, NGT, and PN have not been compared using a population-based database.

Japan established universal health insurance coverage in 1961 [19], with local governments providing payment for < 2% of the population who are on welfare, with exceptions such as accidents covered by automobile liability insurance or worker’s accident compensation [20]. Previous studies using health insurance claims in Japan have been performed to evaluate medical costs [21], vulnerable populations for disasters [22], and population-based incidence of specific diseases [20, 23]. In 2008, Japan reformed health insurance for the population aged ≥ 75 years to support increasing medical expenditures for the older population. The insurance system was managed at the prefectural levels, and 50%, 40%, and 10% of medical expenditures were paid by public funds and healthcare insurance premiums of the working population and that of the older population, respectively [24]. In the Nara Prefecture, the national insurance covered approximately 99% of the 170,000 people aged ≥ 75 years in 2013. The claims data contained information on patient characteristics, diseases during hospital admissions, outpatient treatment, and the date of procedures and death among the prefectural population aged ≥ 75 years. Each dataset could be merged using a patient-matching technique, which was developed for the national health insurance claims in Japan [25]. Therefore, the administrative claims form a population-based cohort data to allow survival analysis from the date of a specific procedure to death, at the prefectural level.

### Aim

This study described the 730-day (2-year) prognoses after initiation of nutrition through GS, NGT, or PN in patients aged ≥ 75 years.

## Methods

### Design

We conducted a population-based cohort study using the national health insurance claims in the Nara Prefecture, Japan.

### Setting

This study was conducted in the Nara Prefecture, Japan. According to Japan’s national census, which is taken every 5 years, the prefectural population was 1.36 million in 2015 with a population density of 370 persons per square kilometer (/km^2^). Of the prefectural population, 180,549 persons were aged ≥ 75 years (13%). For reference, the population and population density in Japan were 127 million and 341 persons/km^2^, respectively, with 13% of the population being ≥ 75 years old, in 2015.

### Participants

This study enrolled patients who were insured by health insurance for the geriatric population aged ≥ 75 years between April 2013 and March 2018. In the dataset, patients who received enteral feeding and PN were classified into GS, NGT, and PN groups using in-hospital claims between April 2014 and March 2016 and were followed up for 730 days (2 years) after initiation.

### Primary GS, secondary GS, NGT, and PN

First, patients who underwent gastrostomies were defined as the GS group. Because of the nature of the claims, this study could not categorize gastrostomies into PEG versus surgical gastrostomies. Second, patients with claims for PN or placements of implantable central venous ports for PN were categorized into the PN group. This study could distinguish placements for PN from those for chemotherapies because these treatments had different codes in the claims data. Third, patients with claims for NGT or placements for enteral feeding were categorized into the NGT group. In addition, the GS group was further categorized as (1) primary GS without any history of preceding NGT or PN within 365 days or (2) secondary GS with preceding NGT or PN within 365 days. This study excluded patients who initiated NGT or PN in an out-of-hospital setting and those who underwent GS several times even in a hospital setting because timing of initiation was unclear in the claims data. In addition, to compare the prognoses of NGT and PN groups without preceding NGT or PN, patients who received NGT or placements before PN were excluded (n = 48). Because some claims for NGT were included in a bundled payment system in in-hospital claims for chronic care and out-of-hospital care, this study preferably enrolled PN before categorizing NGT. Furthermore, patients who underwent GS, NGT, and PN in hospitals outside the Nara Prefecture were excluded to adjust the data according to the hospital area.

### Outcome

This study investigated mortality within 730 days after undergoing a primary GS, secondary GS, NGT, and PN. Under the universal health insurance scheme for the population aged ≥ 75 years, time of death must be claimed at the insurance offices in local governments within 14 days of the patient’s death. Therefore, the information on death can be updated in the claims database as long as the patients are covered by insurance.

### Other variables

The following data were extracted for this study: sex, age, date of death, date that the insurance expired, timing of removal or closure of GS, hospital payment type, i.e., Diagnosis Procedure Combination and Per-Diem Payment System (DPC/PDPS) or non-DPC/PDPS, hospital area, number of hospital beds, and in-hospital disease categories coded by the International Statistical Classification of Diseases and Related Health Problems (ICD)-10. Information on the number of hospital beds was obtained from the public data and official information from each hospital. DPC/PDPS is a prospective payment method for acute inpatient care in Japan [26]. The analyses were adjusted for this variable as an indicator of acute care hospitals relative to non-DPC/PDPS hospitals providing chronic care, psychiatric care, and rehabilitation. In the Nara Prefecture, almost 70 hospitals are located in five medical areas (hospital areas) as follows [27]: Capital City (population density in 2015: 1,301 persons/km^2^), Eastern (319 persons/km^2^), Western (2,044 persons/km^2^), Central (1,561 persons/km^2^), and Southern (31 persons/km^2^) areas. This study also adjusted for hospital area and number of hospital beds, as these could influence the choice of procedures for enteral feeding or PN due to differences in the availability of medical resources or specialists.

Comorbidities were extracted from the disease information in-hospital claims within 365 days before the initiation of nutrition through GS, NGT, and PN. This study identified all diseases recorded in the claims for hospital admissions. The diseases with a “suspicious” flag in the claims datasets were excluded from the comorbidities because they were used to justify diagnostic procedures at non-DPC/PDPS hospitals with a fee-for-service payment system [28]. The comorbidities were categorized by ICD-10 codes for the Charlson Comorbidity Index (CCI) [29, 30], as well as underlying diseases [17]. Total CCI scores were calculated using updated CCI scores in 2011 [31]. Cerebrovascular diseases, myocardial infarction, peripheral vascular disease, peptic ulcers, and diabetes without chronic complications were excluded from the original version of the CCI score [29–31]. In addition, the comorbidities were summarized based on two groups: malignancies and non-malignant diseases. Furthermore, major underlying diseases in the non-malignant diseases group were extracted from in-hospital claims when the patients received GS, NGT, and PN. This study described the major underlying diseases as follows: cerebrovascular disease, neuromuscular disease, dementia, and pneumonia. Table 1 shows the ICD-10 coding and scoring algorithms for the CCI scores and the major underlying diseases.

**Table 1:** ICD-10 coding and scoring algorithms for the Charlson Comorbidity Index

Comorbidities/underlying diseases	ICD-10	Score
**Comorbidities**^a^		
Chronic pulmonary disease	I278; I279; J40-J47; J60-J67; J684; J701; J703	1
Rheumatologic disease (connective tissue disease)	M05; M06; M315; M32; M33; M34; M351; M353; M360	1
Diabetes with chronic complications (diabetes with end-organ damage)	E102-E105; E107; E112-E115; E117; E122-E125; E127; E132-E135; E137; E142-E145; E147	1
Renal disease (moderate or severe renal disease)	I120; I131; N032-N037; N052-N057; N18; N19; N250; Z490; Z491; Z492; Z940; Z992	1
Congestive heart failure	I110; I130; I132; I255; I420; I425-I429; I43; I50; P290	2
Dementia	F00-F03; F051; G30; G311	2
Mild liver disease	B18; K700-K703; K709; K713-K715; K717; K73; K74; K760; K762-K764; K768; K769; Z944	2
Hemiplegia or paraplegia	G041; G114; G801; G802; G81; G82; G830-G834; G839	2
Any malignancy including leukemia and lymphoma^b^	C00-C26; C30-C34; C37-C41; C43; C45-C58; C60-C76; C81-C85; C88; C90-C97	2
Moderate or severe liver disease	I850; I859; I864; I982; K704; K711; K721; K729; K765; K766; K767	4
AIDS/HIV	B20-B22; B24; Z21	4
Metastatic solid tumor^b^	C77-C80	6
**Major underlying diseases**^**c**^		
Cerebrovascular disease	G45; G46; H340; I60-I69	-
Neuromuscular disease	F023; G10-13; G20-23; G35-37; G61; G700; G71; G903; G91	-
Dementia	F00-F03; F051; G30; G311	-
Pneumonia	J120-189, J690	-

^a^Comorbidities for the Charlson Comorbidity Index were extracted from the disease information in-hospital claims within 365 days before the initiation of gastrostomy (GS), nasogastric tube feeding (NGT), and parenteral nutrition (PN)

^b^Malignancies

^c^Major underlying diseases were extracted from in-hospital claims when the patients initially received GS, NGT, and PN

*ICD* International Statistical Classification of Diseases and Related Health Problems

### Statistical analysis

Mortality rates at 30 days, 180 days, 365 days, and 730 days after the initiation of nutrition through GS, NGT, and PN were calculated in patients with or without malignant disease. Because malignancies were considered likely to influence both decisions to initiate the procedures and patients’ prognoses, this study stratified the participants into two groups: with or without malignancies. Data were censored at the time the last claims were made in cases where patients were lost to follow-up. Patients who were alive 730 days after GS, NGT, and PN were censored in the overall survival analysis. In addition, Chi-squared test was performed to compare patient characteristics among the secondary GS, primary GS, NGT, and PN groups. Furthermore, Cox regression analyses were performed to compare the survival rates of the four groups in case of malignancies or non-malignant diseases. In the multivariable analyses, age, sex, hospital information (DPC/PDPS, total beds, areas), and total CCI scores were adjusted as potential confounders with respect to mortality. In addition, Kaplan-Meier analyses followed by log-rank tests were performed to compare survival periods between the secondary GS and primary GS groups. All statistical analyses were performed using IBM SPSS Statistics for Windows, version 24 (IBM Corp., Armonk, NY, USA). A *p*-value < 0.05 was considered statistically significant.

## Results

**Fig. 1:**
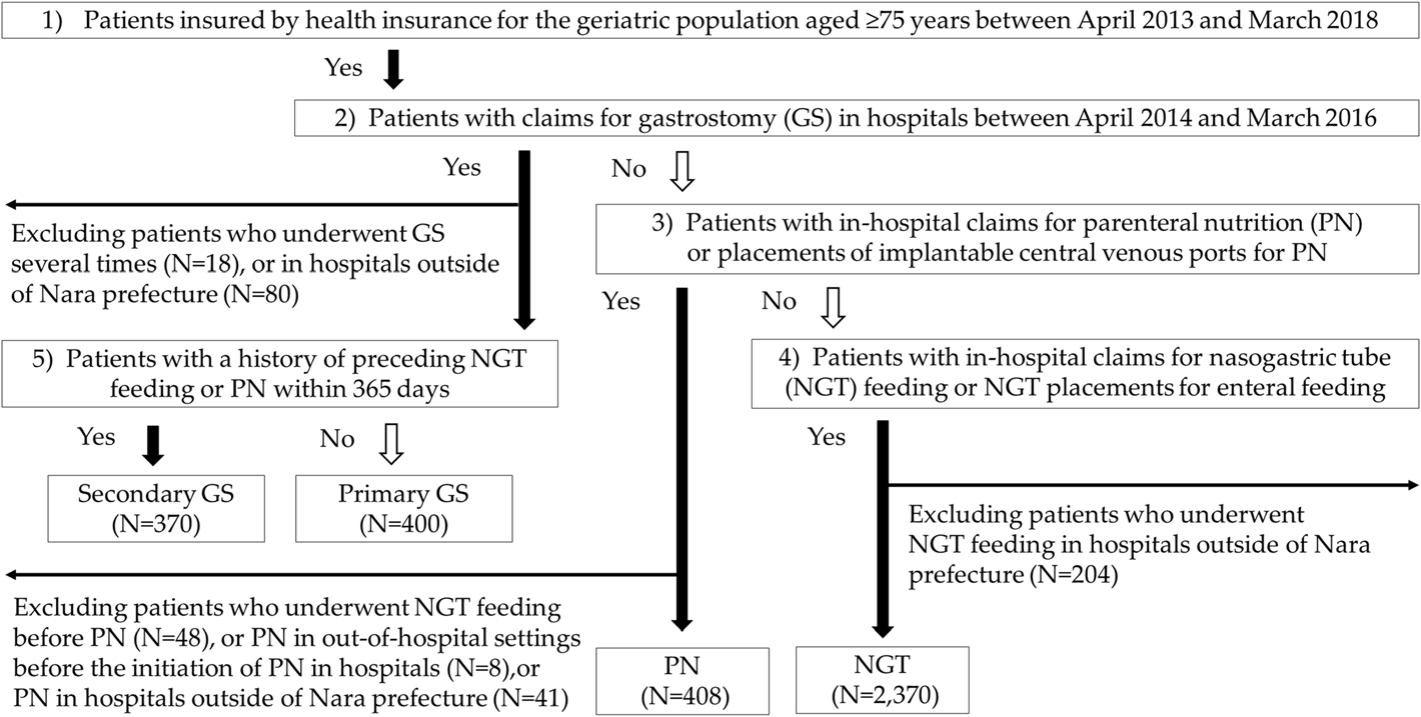
Flow chart showing the enrolment of patients who underwent enteral feeding and parenteral nutrition

This study enrolled 3,548 patients who underwent enteral feeding or PN during hospital admissions between April 2014 and March 2016. Of the 3,548 patients, 3,488 (98%) were followed up until death or censored at 730 days after the initiation of enteral feeding or PN. The patients were categorized into four groups: secondary GS (*n* = 370), primary GS (*n* = 400), NGT (*n* = 2,370), and PN (*n* = 408). Figure 1 shows a flowchart depicting the enrolment of patients in the study. Out of 370 patients in the secondary GS group, 356 (96%) had a history of preceding NGT, while 14 (4%) had a history of preceding PN or both PN and NGT. The intervals between the first NGT and secondary GS were as follows: <30 days (42%), 30–89 days (35%), 90–179 days (12%), and ≥ 180 days (11%). Among 770 patients who underwent secondary or primary GS, 13 patients (2%) had claims for closures or removals of GS within 730 days after the procedures. In total, 98% of the participants were followed up until death or censored at 730 days after initiation of enteral feeding or PN.

Table 2 shows the patients’ characteristics considering the secondary GS, primary GS, NGT, and PN. The mean ages (standard deviation) of the patients who received secondary GS, primary GS, NGT, and PN were 83.1 (5.1), 84.1 (5.4), 83.2 (4.9), and 81.6 (4.5) years in patients with malignancies and 85.2 (5.2), 85.7 (5.5), 85.2 (5.8), and 86.4 (6.1) in those with non-malignant diseases, respectively. Patients in PN groups with malignancies were younger than those in the secondary GS, primary GS, and NGT groups. Total CCI scores for the NGT group were lower than for other groups in the non-malignant group. In addition, the secondary GS and NGT were more frequently performed compared to PN in patients with cerebrovascular diseases in the non-malignant diseases group. Moreover, small and non-DPC/PDPS hospitals tended to perform GS more often than NGT. Regarding hospital region, secondary and primary GS were favored in the Western area and NGT was frequent in the Eastern area.

**Table 2:** Characteristics of patients who received four types of enteral feeding and parenteral nutrition

	Non-malignancies					Malignancies				
	GS		NGT	PN		GS		NGT	PN	
	Primary	Secondary				Primary	Secondary			
	*N* = 352	*N* = 331	*N* = 2042	*N* = 135	p*	*N* = 48	*N* = 39	*N* = 328	*N* = 273	p*
Female, n (%)	215 (61)	200 (60)	1143 (56)	81 (60)	0.155	17 (35)	14 (36)	107 (33)	122 (45)	0.024
Age ≥ 85 years, n (%)	207 (59)	177 (53)	1077 (53)	82 (61)	0.069	25 (52)	14 (36)	125 (38)	65 (24)	< 0.001
Total CCI score, n (%)					< 0.001					0.244
0–1	132 (38)	119 (36)	910 (45)	49 (36)		0 (0)	0 (0)	0 (0)	0 (0)	
2–3	132 (38)	132 (40)	794 (39)	50 (37)		22 (46)	11 (28)	134 (41)	99 (36)	
≥ 4	88 (25)	80 (24)	338 (17)	36 (27)		26 (54)	28 (72)	194 (59)	174 (64)	
Major underlying diseases, n (%)										
Cerebrovascular disease	58 (16)	95 (29)	598 (29)	<10^a^	< 0.001	0 (0)	<10^a^	39 (12)	<10^a^	< 0.001
Neuromuscular diseases	22 (6)	24 (7)	68 (3)	<10^a^	0.001	0 (0)	<10^a^	<10^a^	<10^a^	0.005
Dementia	14 (4)	<10^a^	38 (2)	0 (0)	0.022	0 (0)	0 (0)	<10^a^	0 (0)	0.220
Pneumonia	128 (36)	81 (24)	290 (14)	32 (24)	< 0.001	13 (27)	<10^a^	28 (9)	12 (4)	< 0.001
Hospital types, n (%)										
DPC/PDPS	137 (39)	165 (50)	1544 (76)	63 (47)	< 0.001	25 (52)	20 (51)	262 (80)	199 (73)	< 0.001
Hospital beds, n (%)					< 0.001					< 0.001
< 200	200 (57)	165 (50)	399 (20)	60 (44)		20 (42)	14 (36)	40 (12)	65 (24)	
≥ 200	152 (43)	166 (50)	1643 (80)	75 (56)		28 (58)	25 (64)	288 (88)	208 (76)	
Hospital area, n (%)					< 0.001					< 0.001
Capital City	94 (27)	71 (21)	420 (21)	27 (20)		<10^a^	<10^a^	68 (21)	68 (25)	
Eastern	28 (8)	40 (12)	842 (41)	17 (13)		<10^a^	<10^a^	130^c^ (40)	39 (14)	
Western	123 (35)	110 (33)	317 (16)	50^b^ (37)		16 (33)	14 (36)	42 (13)	80 (29)	
Central	83 (24)	77 (23)	386 (19)	40 (30)		14 (29)	<10^a^	82 (25)	66 (24)	
Southern	24 (7)	33 (10)	77 (4)	<10^a^		<10^a^	<10^a^	<10^a^	20 (7)	

*GS* gastrostomy, *NGT* nasogastric tube feeding, *PN* parenteral nutrition, *CCI* Charlson Comorbidity Index, *DPC/PDPS* Diagnosis Procedure Combination and Per-Diem Payment System

*Chi-square test

^a^When the number of the patients was less than 10 (except 0), the value was not disclosed, according to the cell size suppression policy of the national health insurance data

^b^In the non-malignancy category, the number of PN in the Western hospital area was rounded to prevent back-calculation of the number of PN in the Southern area

^c^In the malignancy category, the number of NGT in the Eastern hospital area was rounded to prevent back-calculation of the number of NGT in the Southern area

**Fig. 2:**
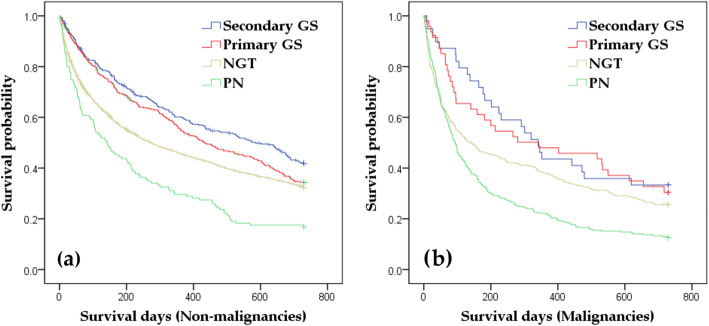
Kaplan-Meier curves for 730-day mortality after initiation of enteral feeding or PN in patients with non-malignant diseases (2**a**) and malignancies (2**b**) Data were censored when the patients were uninsured by the national health insurance. PN: parenteral nutrition, NGT: nasogastric tube feeding, primary GS: gastrostomy without preceding NGT or PN, Secondary GS: gastrostomy secondary to preceding NGT or PN

During the 2-year follow-up, 2,384 patients died, including 210, 255, 1,570, and 349 deaths in the secondary GS, primary GS, NGT, and PN groups, respectively. Table 3 shows the mortalities in the four groups stratified by malignancy and non-malignant diseases groups at 30 days, 180 days, 365 days, and 730 days after initiation. The Kaplan-Meier survival curves in patients with non-malignant diseases and malignancies are described in Fig. 2a and b, and the Cox regression analyses of the two groups adjusted for age, sex, CCI scores, and hospital information are shown in Table 4. Both in the non-malignancy and malignancy groups, enteral feeding, including secondary GS, primary GS, and NGT, were associated with significantly lower mortality rates than PN. In the non-malignant diseases group, secondary GS also showed a significantly lower hazard ratio (HR) than NGT. In addition, the multivariable analyses indicated that older age and high CCI score were significantly related to death within 730 days after initiation of enteral feeding or PN, regardless of malignancy. Furthermore, in patients with non-malignant disease, non-DPC/PDPS hospital and region differences were significantly related to death within 730 days after initiation.

**Table 3:** Mortality within 730 days after initiating primary GS, secondary GS, NGT, and PN

	GS		NGT	PN
	Primary	Secondary		
Mortality (Death^a^/At risk^b^)				
Non-malignant diseases				
30 days	7% (25/348)	7% (22/328)	17% (336/2029)	21% (29/135)
180 days	31% (106/343)	27% (87/324)	44% (866/1990)	56% (75/135)
365 days	46% (156/339)	40% (129/320)	55% (1080/1978)	70% (95/135)
730 days	66% (223/338)	58% (184/316)	68% (1328/1967)	83% (112/135)
Malignancies				
30 days	- (<10^c^/47)	- (<10^c^/39)	24% (80/327)	21% (57/273)
180 days	39% (18/46)	31% (12/39)	54% (176/326)	67% (183/272)
365 days	52% (24/46)	56% (22/39)	62% (202/326)	78% (212/272)
730 days	71% (32/45)	67% (26/39)	74% (242/326)	87% (237/272)

*GS* gastrostomy, *NGT* nasogastric tube feeding, *PN* parenteral nutrition

^a^Number of participants who were dead at each point in time

^b^Number of participants excluding non-death censors at each point in time

^c^When the number of the patients was less than 10 (except 0), the value was not disclosed, according to the cell size suppression policy of the national health insurance data

**Table 4:** Cox regression analysis after initiation of enteral feeding and parenteral nutrition

	Non-malignancies					Malignancies				
	Hazard ratio	95% CI			*p*	Hazard ratio	95% CI			*p*
Gender										
Male	1.61	1.47	–	1.77	< 0.001	1.16	0.96	–	1.39	0.124
Female	Reference	–		–	–	Reference	–		–	–
Age										
≥ 85	1.48	1.34	–	1.62	< 0.001	1.43	1.18	–	1.74	< 0.001
76–84	Reference	–		–	–	Reference	–		–	–
Comorbidities (CCI)										
0–1	0.86	0.75	–	0.99	0.029	–	–		–	–
2–3	1.07	0.94	–	1.22	0.286	0.68	0.57	–	0.82	< 0.001
≥ 4	Reference	–		–	–	Reference	–		–	–
Hospital type										
DPC	0.85	0.75	–	0.95	0.007	0.84	0.67	–	1.05	0.121
Non-DPC	Reference	–		–	–	Reference	–		–	–
Hospital beds										
≥ 200	1.03	0.91	–	1.17	0.654	1.04	0.79	–	1.36	0.79
< 200	Reference	–		–	–	Reference	–		–	–
Hospital area										
Capital city	0.68	0.55	–	0.86	0.001	1.13	0.73	–	1.77	0.583
Eastern	0.62	0.50	–	0.78	< 0.001	1.23	0.77	–	1.98	0.392
Western	0.82	0.66	–	1.03	0.084	1.13	0.71	–	1.78	0.61
Central	0.70	0.55	–	0.87	0.002	1.13	0.73	–	1.75	0.581
Southern	Reference	–		–	–	Reference	–		–	–
Enteral feeding and parenteral nutrition										
Secondary GS	0.43	0.34	–	0.54	< 0.001	0.42	0.28	–	0.64	< 0.001
Primary GS	0.51	0.40	–	0.64	< 0.001	0.46	0.32	–	0.68	< 0.001
NGT	0.71	0.58	–	0.87	0.001	0.66	0.54	–	0.80	< 0.001
PN	Reference	–		–	–	Reference	–		–	–

*CI* confidence interval, *CCI* Charlson Comorbidity Index, *DPC/PDPS* Diagnosis Procedure Combination and Per-Diem Payment System, *GS* gastrostomy, *NGT* nasogastric tube feeding, *PN* parenteral nutrition

**Fig. 3:**
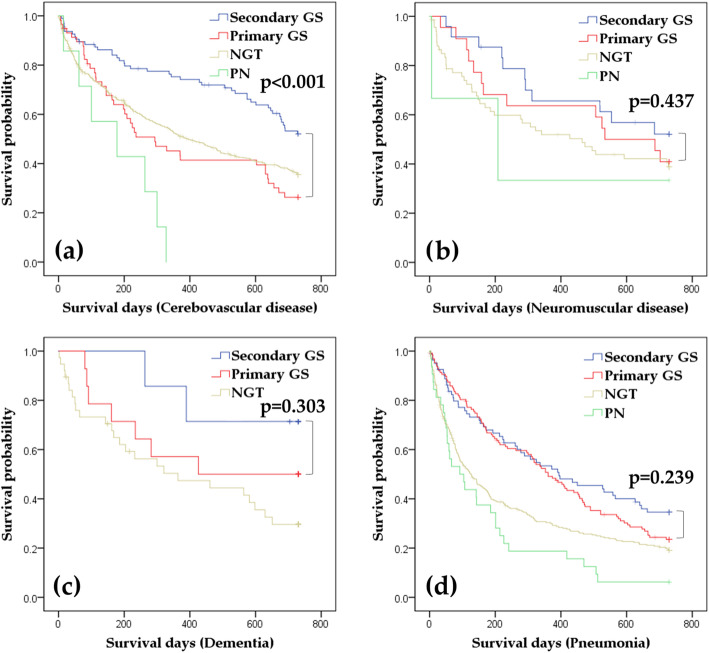
Kaplan-Meier curves for 730-day mortality after initiation of enteral feeding or PN in patients with non-malignant diseases: subgroup analysis of cerebrovascular disease (3**a**), neuromuscular disease (3**b**), dementia (3**c**), and pneumonia (3**d**) Log-rank tests were performed between primary GS and secondary GS. Data were censored when the patients were uninsured by the national health insurance. PN: parenteral nutrition, NGT: nasogastric tube feeding, primary GS: gastrostomy without preceding NGT or PN, Secondary GS: gastrostomy secondary to preceding NGT or PN

The non-malignant group was divided into the following subgroups: cerebrovascular disease (Fig. 3a), neuromuscular disease (Fig. 3b), dementia (Fig. 3c), and pneumonia (Fig. 3d). The subgroup analysis by log-rank test revealed that the secondary GS group had significantly lower mortality than the primary GS group among patients admitted for cerebrovascular disease (*p* < 0.001), whereas no significant differences in mortalities were found with neuromuscular disease, dementia, and pneumonia.

## Discussion

This study evaluated survival following initiation of enteral feeding and PN in a population aged 75 years and older. Overall, the 2-year mortality after initiation of feeding through secondary GS and primary GS, NGT, and PN ranged from 58–87%, and patients with malignancies tended to have higher mortality rates. In addition, our findings indicated the prognoses could be influenced by patient characteristics, comorbidities, and hospital information. Therefore, enteral feeding and PN should be carefully initiated based on the estimated prognosis, each patient’s condition, and the background of the patient.

This study indicated the survival benefits and limitations of enteral feeding and PN for the older population who cannot feed themselves. A randomized controlled trial in the United Kingdom demonstrated the efficacy of early tube feeding and subsequent reduction in mortality among patients with a history of dysphagic stroke [3]. Similarly, timely initiation of NGT and secondary GS could help reduce mortality in older patients who require enteral feeding in patients with cerebrovascular disease. These findings suggest the importance of early nutritional interventions for malnutrition conditions in older patients. However, in the group with cerebrovascular disease, only 95 patients in the secondary GS group had their enteral feeding switched from NGT to GS compared with 598 patients who underwent NGT feeding without subsequent GS. This fact indicated that most patients with cerebrovascular disease and those who underwent NGT might have continued or stopped feeding without switching to secondary GS over the two years. In addition, this study revealed that only 2% of patients who underwent GS experienced closures or removals. The results might indicate difficulties in weaning patients aged over 75 years from GS. In Japan, the number of patients who undergo PEG tube feeding has been decreasing since its peak in 2007. This might be related to the increase in public awareness regarding quality-of-life or end-of-life care [32]. Enteral feeding, especially PEG, has been discussed as a part of life-sustaining treatments at the end of life [33, 34]. Therefore, healthcare professionals, together with patients and their surrogates, should carefully consider the indications and types of enteral feeding in view of the benefits as well as the religious and cultural beliefs of the decision-makers.

The Cox regression analyses showed that the 2-year mortality rate after the initiation of secondary GS was the lowest compared to that of PN both in the non-malignant and malignancy groups. A previous study that included older patients in Japan, retrospectively compared survival time among patients with dysphagia who required PEG and PN [35]. They found that the survival period was significantly longer in the PEG group (median, 317 days) than that in the PN group (195 days). Although the study excluded patients with advanced cancer [35], our study demonstrated better prognoses within 2 years for enteral nutrition than for PN in patients with and without malignant diseases. Moreover, long-term PN places patients at risk for catheter-related blood-stream infections and bacterial translocation [35–37]. However, in the multivariable analysis, some factors that could influence clinical decisions on nutrition for patients receiving end-of-life care were not adjusted. For instance, PN might be chosen for short-term nutrition over invasive enteral feeding in such situations. Therefore, this study suggested that PN should not be an alternative to long-term enteral feeding for patients with normal gastrointestinal functions among those who are expected to have year-long prognoses.

A substantial strength of this study is that a 2-year survival analysis was performed using population-based claims data that followed approximately 98% of the patients from the initiation of secondary GS, primary GS, NGT, and PN until death. In addition, this study evaluated patients who were newly initiated with enteral feeding or PN such as primary and secondary GS. Therefore, the results of this study could be useful for explaining the effectiveness and limitations of enteral feeding and PN. In contrast, several limitations of this study should be acknowledged. First, this study did not evaluate validated nutritional indicators, such as the Mini Nutritional Assessment (MNA) or the Geriatric Nutritional Risk Index (GNRI), to detect malnutrition in hospitalized older patients [38]. For example, the claims data did not list MNA information, comprising decreased food intake, weight loss, comorbidities, and physical information such as body mass index, mid-arm circumference, and calf circumstance [38, 39], or GNRI data, including serum albumin, bodyweight, and height [39]. Although the multivariable Cox regression analysis was adjusted for CCI scores using in-hospital claims within 365 days to reduce bias associated with comorbidities in assessing mortality rates associated with the four groups, these unmeasured factors remain and may result in residual confounding bias. Second, this study did not evaluate the continuity of NGT and PN. Since claims of NGT and PN were included in the bundled payment system for medical supervision charges in out-of-acute hospital settings in Japan, this study could not count the claims after hospital discharge. In addition, this study excluded patients who underwent PN with preceding NGT. These limitations could be biases in the comparisons of the prognoses results between PN and NGT. Because of the nature of the claims this study focused on the survival intervals between the initiation of enteral feeding and PN during hospital admission and death in all the settings, regardless of whether their uses were continued. Considering that previous studies on prognoses of NGT or PN evaluated the periods between initiation and death [16, 35], our findings are comparable with those of these studies. However, to address these concerns, further research is needed to clarify the prognoses associated with enteral feeding and PN in patients with different conditions and circumstances.

## Conclusions

The 2-year mortalities of patients aged ≥ 75 years who received enteral feeding or PN ranged from 58–87%. Enteral feeding including secondary GS, primary GS, and NGT had a better 2-year prognosis than PN in older patients with and without malignant disease. If older patients with cerebrovascular disease are found to be suitable for long-term enteral feeding, switching from partial NGT or PN to secondary GS might provide longer survival than with PN. These findings should be cautiously interpreted with respect to patient characteristics such as comorbidities, severity of condition, and willingness to receive feeding.

## Data Availability

The datasets generated during the current study are not publicly available owing to the publication policy of the Nara Prefecture but are available from the corresponding author on reasonable request subject to approval from the Nara Prefecture.

## Abbreviations

GS: Gastrostomy
NGT: Nasogastric tube
PN: Parenteral nutrition
PEG: Percutaneous endoscopic gastrostomy
CCI: Charlson Comorbidity Index
DPC/PDPS: Diagnosis Procedure Combination and Per-Diem Payment System
ICD: International Statistical Classification of Diseases and Related Health Problems
MNA: Mini Nutritional Assessment
GNRI: Geriatric Nutritional Risk Index
HR: Hazard ratio
